# Crystal structure and Hirshfeld surface analysis of aqua­bis­(nicotinamide-κ*N* ^1^)bis­(2,4,6-tri­methyl­benzoato-κ*O*)zinc

**DOI:** 10.1107/S2056989017011690

**Published:** 2017-08-21

**Authors:** Tuncer Hökelek, Gülçin Şefiye Aşkın, Safiye Özkaya, Hacali Necefoğlu

**Affiliations:** a Department of Physics, Hacettepe University, 06800 Beytepe, Ankara, Turkey; b Department of Chemistry, Kafkas University, 36100 Kars, Turkey; c Department of Chemistry, Kafkas University, 36100 Kars, Turkey, International Scientific Research Centre, Baku State University, 1148 Baku, Azerbaijan

**Keywords:** crystal structure, zinc, transition metal complex of benzoic acid and nicotinamide derivatives

## Abstract

The asymmetric unit of the title complex, [Zn(C_10_H_11_O_2_)_2_(C_6_H_6_N_2_O)_2_(H_2_O)], contains one half of the complex mol­ecule, and the Zn^II^ cation and the water O atom lie on a twofold rotation axis. The Zn^II^ cation is coordinated by two carboxyl­ate O atoms of the two symmetry-related 2,4,6-tri­methyl­benzoate (TMB) anions and by the water O atom at distances of 2.0311 (16) and 2.076 (2) Å to form a slightly distorted trigonal–planar arrangement, while the distorted trigonal–bipyramidal coordination sphere is completed by the two pyridine N atoms of the two symmetry-related monodentate nicotinamide (NA) ligands at distances of 2.2066 (19) Å in the axial positions. In the crystal, mol­ecules are linked *via* inter­molecular N—H⋯O and O—H⋯O hydrogen bonds with *R*_2_^2^(12), *R*_3_^3^(10) and *R*_3_^3^(16) ring motifs, forming a double-column structure running along the *c*-axis direction. The Hirshfeld surface analysis of the crystal structure indicates that the most important contributions for the crystal packing are from H⋯H (58.4%), H⋯C/C⋯H (20.3%) and H⋯O/O⋯H (18.3%) inter­actions.

## Abstract 2

In the title Zn complex, the Zn^II^ cation is five-coordinated in a distorted trigonal–bipyramidal geometry. The Hirshfeld surface analysis of the crystal structure indicates that the most important contributions for the crystal packing are from H⋯H (58.4%), H⋯C/C⋯H (20.3%) and H⋯O/O⋯H (18.3%) inter­actions.

## Chemical context   

Nicotinamide (NA) is one form of niacin. A deficiency of this vitamin leads to loss of copper from the body, known as pellagra disease. Victims of pellagra show unusually high serum and urinary copper levels (Krishnamachari, 1974 ▸). The NA ring is the reactive part of nicotinamide adenine dinucleotide (NAD) and its phosphate (NADP), which are the major electron carriers in many biological oxidation-reduction reactions (You *et al.*, 1978 ▸). A nicotinic acid derivative, *N*,*N*-di­ethyl­nicotinamide (DENA), is an important respiratory stimulant (Bigoli *et al.*, 1972 ▸).

The transition metal complexes with ligands of biochemical inter­est as imidazole and some *N*-protected amino acids show inter­esting physical and/or chemical properties, through which they may find applications in biological systems (Antolini *et al.*, 1982 ▸). Crystal structures of metal complexes with benzoic acid derivatives have been reported extensively because of the varieties of the coordination modes [for example, Co and Cd complexes with 4-amino­benzoic acid (Chen & Chen, 2002 ▸)]. The structures of some mononuclear complexes obtained from the reactions of transition metal(II) ions with nicotinamide (NA) and some benzoic acid derivatives as ligands, *e.g.* [Zn(C_7_H_5_O_3_)_2_(C_6_H_6_N_2_O)_2_] [(II); Necefoğlu *et al.*, 2002 ▸], [Mn(C_7_H_4_ClO_2_)_2_(C_10_H_14_N_2_O)_2_(H_2_O)_2_] [(III); Hökelek *et al.*, 2008 ▸], [Zn(C_8_H_8_NO_2_)_2_(C_6_H_6_N_2_O)_2_]·H_2_O [(IV); Hökelek *et al.*, 2009*a* ▸], [Mn(C_9_H_10_NO_2_)_2_(C_6_H_6_N_2_O)(H_2_O)_2_] [(V); Hökelek *et al.*, 2009*b* ▸], [Ni(C_7_H_4_ClO_2_)_2_(C_6_H_6_N_2_O)_2_(H_2_O)_2_] [(VI); Hökelek *et al.*, 2009*c* ▸] and [Zn(C_7_H_4_BrO_2_)_2_(C_6_H_6_N_2_O)_2_(H_2_O)_2_] [(VII); Hökelek *et al.*, 2009*d* ▸], have been determined previously. The structure determination of the title compound, [Zn(C_10_H_11_O_2_)_2_(C_6_H_6_ON_2_)_2_(H_2_O)] (I), a zinc complex with two 2,4,6-tri­methyl­benzoate (TMB) and two nicotinamide (NA) ligands and one coordinating water mol­ecule, was undertaken in order to compare the results obtained with those reported previously. In this context, we synthesized the title compound and report herein its crystal and mol­ecular structures along with the Hirshfeld surface analysis.
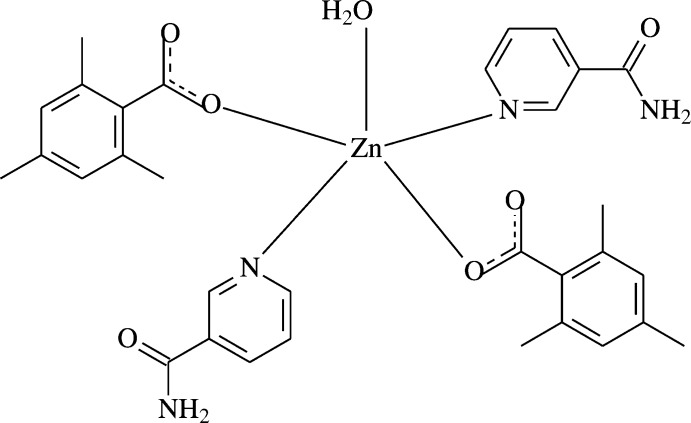


## Structural commentary   

**Figure 1:**
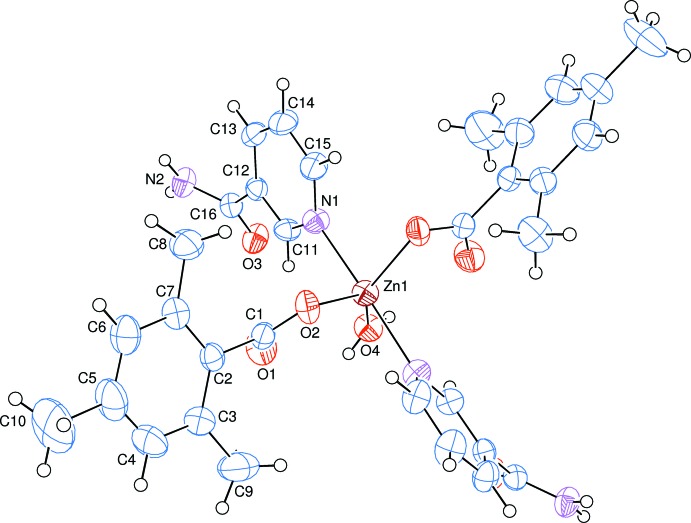
The mol­ecular structure of the title complex, with the atom-numbering scheme. Unlabelled atoms are related to labelled ones by the symmetry operation (1 − *x*, *y*, 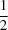 − *z*). Displacement ellipsoids are drawn at the 40% probability level.

The asymmetric unit of the crystal structure of the mononuclear title complex contains one Zn^II^ cation (site symmetry 2), one 2,4,6-tri­methyl­benzoate (TMB) anion and one nicotinamide (NA) mol­ecule together with one water mol­ecule (point group symmetry 2), all ligands coordinating in a monodentate manner (Fig. 1 ▸). The Zn^II^ cation is penta-coordinated *via* two nitro­gen atoms of NA and two oxygen atoms of TMB anions and one oxygen atom of the water mol­ecule. The two carboxyl­ate O atoms [O2 and O2^i^; symmetry code: (i) 1 − *x*, *y*, 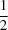 − *z*] of the two symmetry-related monodentate TMB anions and the coordinating water O atom (O4) are at distances of 2.0311 (16) and 2.076 (2) Å, respectively, around the Zn1 atom and form a slightly distorted triangular planar arrangement. The sum of the bond angles O2—Zn1—O2^i^ [95.38 (9)°], O2—Zn1—O4 [132.31 (5)°] and O2—Zn1—O4^i^ [132.31 (5)°] in the basal plane around Zn^II^ cation is 360°. This confirms the presence of the Zn^II^ cation with very slight deviation from the basal plane. The slightly distorted trigonal–bipyramidal coordination sphere is completed by the two pyridine N atoms (N1 and N1^i^) of the two symmetry-related monodentate NA ligands at distances of 2.2066 (19) Å in the axial positions. The index of trigonality τ [where τ = (β - α)/60, in which α and β are the two largest coordination angles; Addison *et al.* (1984 ▸)] was calculated as 0.65 by taking N1—Zn1—N1 as β [171.42 (8)°] and O2—Zn1—O4 as α [132.31 (5)°]. In general, τ = 0 for an ideal square pyramidal and τ = 1 for an ideal trigonal–pyramidal geometry. In the present case, the obtained τ value is slightly closer to a trigonal–pyramidal geometry.

The near equalities of the C1—O1 [1.240 (3) Å] and C1—O2 [1.259 (3) Å] bonds in the carboxyl­ate groups indicate delocalized bonding arrangements rather than localized single and double bonds. The O2—C1—O1 bond angle [121.8 (2)°] seems to be slightly decreased than that present in a free acid [122.2°], in which the O2—C1—O1 bond angle may be compared with the corresponding values of 123.5 (2) and 120.4 (2)° in (II), 125.2 (5)° in (III), 119.2 (3) and 123.8 (2)° in (IV), 123.6 (3) and 119.4 (3)° in (V), 124.4 (2)° in (VI) and 124.3 (2)° in (VII), where the benzoate ions coordinate to the metal atoms only monodentately in (III), (VI) and (VII), and both monodentately and bidentately in (II), (IV) and (V). The Zn1 atom lies 0.0817 (1) Å above of the planar (O1/O2/C1) carboxyl­ate group. In the TMB anion, the carboxyl­ate group is twisted away from the attached benzene C2–C7 ring by 61.32 (14)°, while the benzene ring and the pyridine N1/C11–C15 ring are oriented at a dihedral angle of 81.90 (8)°.

## Supra­molecular features   

**Figure 2:**
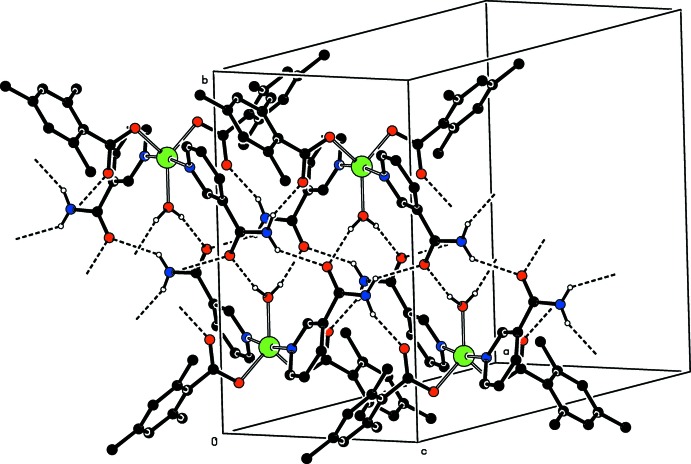
Part of the crystal structure. O—H_coordW_⋯O_NA_, N—H_NA_⋯O_c_ and N—H_NA_⋯O_NA_ (coordW = coordinating water, c = carboxyl­ate and NA = nicotinamide) hydrogen bonds, enclosing 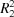
(12), 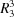
(10) and 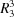
(16) ring motifs, are shown as dashed lines. Non-bonding H atoms have been omitted for clarity.

In the crystal, the NH_2_ group links to the non-coordinating carboxyl­ate and NA oxygen atoms *via* inter­molecular N—H⋯O hydrogen bonds, and the water mol­ecule links to the NA oxygen atoms *via* inter­molecular O—H⋯O hydrogen bonds (Table 1 ▸). These hydrogen bonds, enclosing 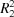
(12), 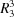
(10) and 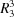
(16) ring motifs, link the mol­ecules into a network consisting of a double-column structure running along the *c*-axis direction (Fig. 2 ▸). No significant π–π, C—H⋯π or C—H⋯O inter­actions are observed.

## Hirshfeld surface analysis   

**Figure 3:**
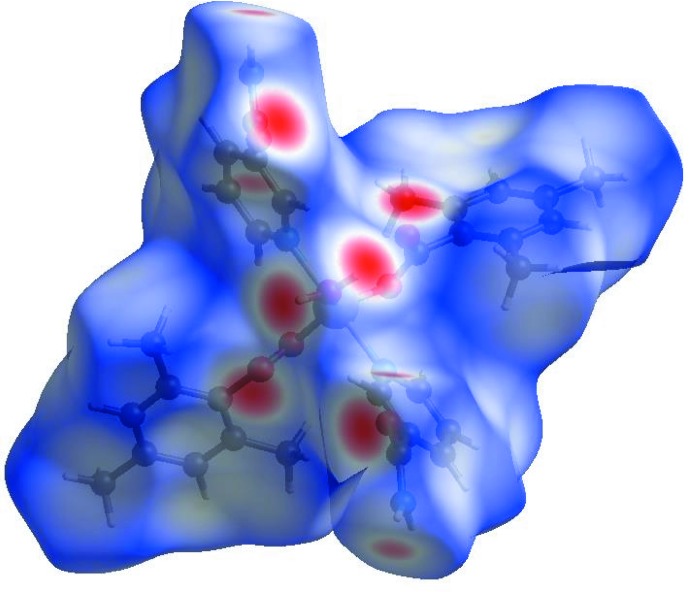
View of the three-dimensional Hirshfeld surface of the title complex plotted over *d*_norm_ in the range −0.6568 to 1.4993 a.u.

**Figure 4:**
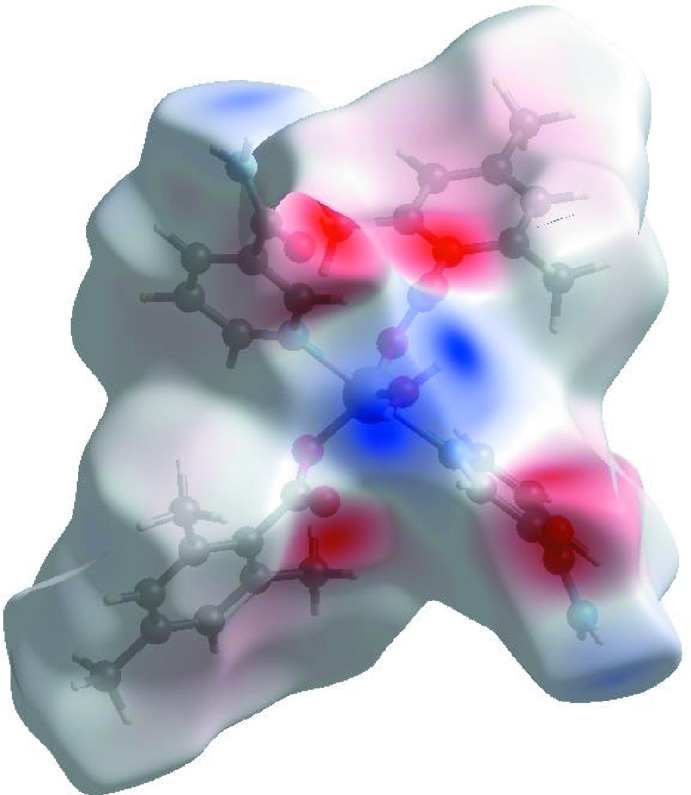
View of the three-dimensional Hirshfeld surface of the title complex plotted over electrostatic potential energy in the range −0.1036 to 0.2354 a.u. using the STO-3G basis set at the Hartree–Fock level of theory. N—H⋯O and O—H⋯O hydrogen-bond donors and acceptors are viewed as blue and red regions around the atoms corresponding to positive and negative potentials, respectively.

**Figure 5:**
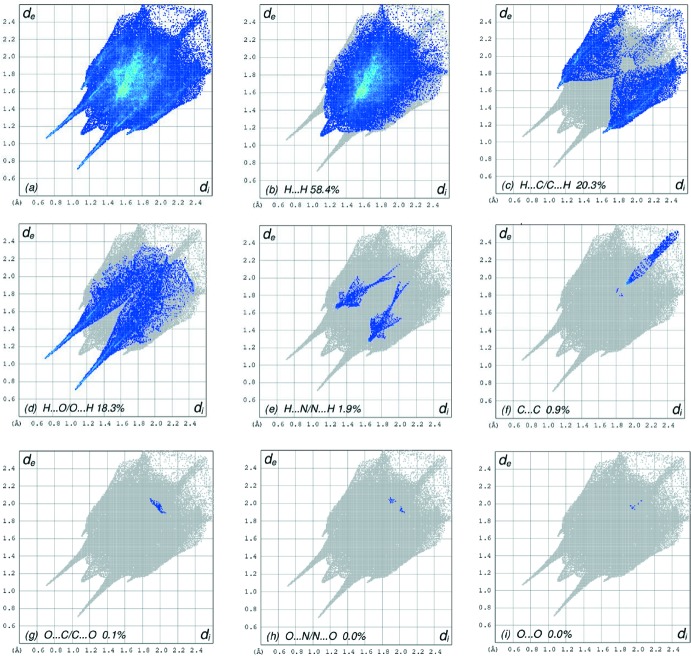
The full two-dimensional fingerprint plots for the title complex, showing (*a*) all inter­actions, and delineated into (*b*) H⋯H, (*c*) H⋯C/C⋯H, (*d*) H⋯O/O⋯H, (*e*) H⋯N/N⋯H, (*f*) C⋯C, (*g*) O⋯C/C⋯O, (*h*) O⋯N/N⋯O and (*i*) O⋯O inter­actions. The *d*_i_ and *d*_e_ values are the closest inter­nal and external distances (in Å) from given points on the Hirshfeld surface contacts.

A Hirshfeld surface (HS) analysis (Hirshfeld, 1977 ▸; Spackman & Jayatilaka, 2009 ▸) was carried out by using *Crystal Explorer 17.5* (Turner *et al.*, 2017 ▸) in order to visualize the inter­molecular inter­actions in the crystal of the title complex. In the HS plotted over *d*_norm_ (Fig. 3 ▸), the white surface indicates contacts with distances equal to the sum of van der Waals radii, and the red and blue colours indicate distances shorter (in close contact) or longer (distant contact) than the van der Waals radii, respectively (Venkatesan *et al.*, 2016 ▸). The bright-red spots appearing near atoms O1, O3, H21, H22 and H41 indicate their role as the respective donors and acceptors in the dominant O—H⋯O and N—H⋯O hydrogen bonds. These O and H atoms also appear as blue and red regions, respectively, corresponding to positive and negative potentials on the HS mapped over electrostatic potential (Spackman *et al.*, 2008 ▸; Jayatilaka *et al.*, 2005 ▸) as shown in Fig. 4 ▸. The blue regions indicate the positive electrostatic potential (hydrogen bond donors), while the red regions indicate the negative electrostatic potential (hydrogen bond acceptors). The overall two-dimensional fingerprint plot and those delineated into H⋯H, H⋯C/C⋯H, H⋯O/O⋯H, H⋯N/N⋯H, C⋯C, O⋯C/C⋯O, O⋯N/N⋯O and O⋯O contacts (McKinnon *et al.*, 2007 ▸) are illustrated in Fig. 5 ▸ *a*–*i*, respectively, together with their relative contributions to the Hirshfeld surface. The most important inter­action is H⋯H contributing 58.4% to the overall crystal packing, which is reflected in Fig. 5 ▸ *b* as widely scattered points of high density due to the large hydrogen content of the mol­ecule. In the absence of C—H⋯π inter­actions in the crystal, the pair of characteristic wings resulting in the fingerprint plot delineated into H⋯C/C⋯H contacts with 20.3% contribution to the HS, Fig. 5 ▸ *c*, and the pair of thin edges at *d*_e_ + *d*_i_ ∼2.9 Å result from short inter­atomic H⋯C/C⋯H contacts. In the fingerprint plot delineated into H⋯O/O⋯H contacts (Fig. 5 ▸ *d*), the 18.3% contribution to the HS arises from the inter­molecular O—H⋯O hydrogen bonding and is viewed as pair of spikes with the tip at *d*_e_ + *d*_i_ ∼1.9 Å. The short H⋯O/O⋯H contacts are masked by strong O—H⋯O hydrogen bonding in this plot. The H⋯N/N⋯H contacts in the structure with 1.9% contribution to the HS has a symmetrical distribution of points with the tips at *d*_e_ + *d*_i_ ∼2.8 Å arising from the short inter­atomic H⋯N/N⋯H contact (Fig. 5 ▸ *e*). The HSs mapped over shape-index, curvedness and those with the function *d*_norm_ plotted onto the surface are shown for the H⋯H, H⋯C/C⋯H, H⋯O/O⋯H and H⋯N/N⋯H inter­actions are shown in Figs. s1–s3 in the Supporting Information.

## Synthesis and crystallization   

The title compound was prepared by the reaction of ZnSO_4_·7H_2_O (0.72 g, 2.5 mmol) in H_2_O (50 ml) and nicotinamide (0.61 g, 5 mmol) in H_2_O (25 ml) with sodium 2,4,6-tri­methyl­benzoate (0.93 g, 5 mmol) in H_2_O (150 ml) at room temperature. The mixture was set aside to crystallize at ambient temperature for ten weeks, giving colourless single crystals (yield: 1.39 g, 85%). FT–IR: 3396, 3111, 2953, 2919, 2740, 2321, 1947, 1693, 1665, 1621, 1601, 1584, 1445, 1397, 1199, 1113, 1047, 860, 839, 797, 731, 647, 614, 545, 559 cm^−1^.

## Refinement   

The experimental details including the crystal data, data collection and refinement are summarized in Table 2 ▸. The H atom of the water mol­ecule was located in a difference-Fourier map and refined freely. H atoms of the NH_2_ group were also located in a difference Fourier map and the positions were refined with *U*_iso_(H) = 1.5*U*_eq_(N). The C-bound H atoms were positioned geometrically with C—H = 0.93 and 0.96 Å for aromatic and methyl H-atoms, respectively, and refined as riding with *U*_iso_(H) = *k* × *U*_eq_(C), where *k* = 1.5 for methyl H-atoms and *k* = 1.2 for aromatic H-atoms.

## Supplementary Material

Crystal structure: contains datablock(s) I, global. DOI: 10.1107/S2056989017011690/is5478sup1.cif

Structure factors: contains datablock(s) I. DOI: 10.1107/S2056989017011690/is5478Isup2.hkl

Hirsfeld surface plotted over shape-index. DOI: 10.1107/S2056989017011690/is5478sup3.pdf

Hirshfeld surface plotted over curvedness. DOI: 10.1107/S2056989017011690/is5478sup4.pdf

Hirshfeld surfaces plotted over dnorm. DOI: 10.1107/S2056989017011690/is5478sup5.pdf

CCDC reference: 1567723

Additional supporting information:  crystallographic information; 3D view; checkCIF report

## Figures and Tables

**Table 1:** Hydrogen-bond geometry (Å, °)

*D*—H⋯*A*	*D*—H	H⋯*A*	*D*⋯*A*	*D*—H⋯*A*
N2—H21⋯O1^i^	0.91 (3)	1.88 (3)	2.766 (3)	165 (3)
N2—H22⋯O3^ii^	0.87 (3)	2.34 (3)	3.013 (2)	134 (2)
O4—H41⋯O3^iii^	0.81 (3)	1.93 (3)	2.719 (2)	165 (3)

Symmetry codes: (i) 
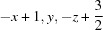
; (ii) 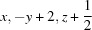; (iii) 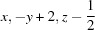.

**Table 2:** Experimental details

Crystal data	
Chemical formula	[Zn(C_10_H_11_O_2_)_2_(C_6_H_6_N_2_O)_2_(H_2_O)]
*M*_r_	654.02
Crystal system, space group	Orthorhombic, *P* *b* *c* *n*
Temperature (K)	296
*a*, *b*, *c* (Å)	23.4004 (5), 15.1685 (4), 9.2353 (3)
*V* (Å^3^)	3278.06 (15)
*Z*	4
Radiation type	Mo *K*α
μ (mm^−1^)	0.80
Crystal size (mm)	0.42 × 0.36 × 0.21

Data collection	
Diffractometer	Bruker SMART BREEZE CCD
Absorption correction	Multi-scan (*SADABS*; Bruker, 2012 ▸)
*T*_min_, *T*_max_	0.730, 0.850
No. of measured, independent and observed [*I* > 2σ(*I*)] reflections	44050, 4104, 3310
*R*_int_	0.036
(sin θ/λ)_max_ (Å^−1^)	0.669

Refinement	
*R*[*F* ^2^ > 2σ(*F* ^2^)], *wR*(*F* ^2^), *S*	0.045, 0.127, 1.08
No. of reflections	4104
No. of parameters	213
H-atom treatment	H atoms treated by a mixture of independent and constrained refinement
Δρ_max_, Δρ_min_ (e Å^−3^)	0.29, −0.32

Computer programs: *APEX2* and *SAINT* (Bruker, 2012 ▸), *SHELXS97* and *SHELXL97* (Sheldrick, 2008 ▸), *ORTEP-3 for Windows* and *WinGX* (Farrugia, 2012 ▸) and *PLATON* (Spek, 2015 ▸).

## supplementary crystallographic information

### Crystal data

**Table:**

[Zn(C_10_H_11_O_2_)_2_(C_6_H_6_N_2_O)_2_(H_2_O)]	*F*(000) = 1368
*M**_r_* = 654.02	*D*_x_ = 1.325 Mg m^−^^3^
Orthorhombic, *P**b**c**n*	Mo *K*α radiation, λ = 0.71073 Å
Hall symbol: -P 2n 2ab	Cell parameters from 9891 reflections
*a* = 23.4004 (5) Å	θ = 2.7–28.4°
*b* = 15.1685 (4) Å	µ = 0.80 mm^−^^1^
*c* = 9.2353 (3) Å	*T* = 296 K
*V* = 3278.06 (15) Å^3^	Block, colourless
*Z* = 4	0.42 × 0.36 × 0.21 mm

### Data collection

**Table:**

Bruker SMART BREEZE CCD diffractometer	4104 independent reflections
Radiation source: fine-focus sealed tube	3310 reflections with *I* > 2σ(*I*)
Graphite monochromator	*R*_int_ = 0.036
φ and ω scans	θ_max_ = 28.4°, θ_min_ = 1.6°
Absorption correction: multi-scan (*SADABS*; Bruker, 2012)	*h* = −29→31
*T*_min_ = 0.730, *T*_max_ = 0.850	*k* = −20→18
44050 measured reflections	*l* = −12→11

### Refinement

**Table:**

Refinement on *F*^2^	Primary atom site location: structure-invariant direct methods
Least-squares matrix: full	Secondary atom site location: difference Fourier map
*R*[*F*^2^ > 2σ(*F*^2^)] = 0.045	Hydrogen site location: inferred from neighbouring sites
*wR*(*F*^2^) = 0.127	H atoms treated by a mixture of independent and constrained refinement
*S* = 1.08	*w* = 1/[σ^2^(*F*_o_^2^) + (0.0569*P*)^2^ + 1.7904*P*] where *P* = (*F*_o_^2^ + 2*F*_c_^2^)/3
4104 reflections	(Δ/σ)_max_ = 0.001
213 parameters	Δρ_max_ = 0.29 e Å^−^^3^
0 restraints	Δρ_min_ = −0.32 e Å^−^^3^

### Special details

**Table:**

Geometry. All esds (except the esd in the dihedral angle between two l.s. planes) are estimated using the full covariance matrix. The cell esds are taken into account individually in the estimation of esds in distances, angles and torsion angles; correlations between esds in cell parameters are only used when they are defined by crystal symmetry. An approximate (isotropic) treatment of cell esds is used for estimating esds involving l.s. planes.
Refinement. Refinement of F^2^ against ALL reflections. The weighted R-factor wR and goodness of fit S are based on F^2^, conventional R-factors R are based on F, with F set to zero for negative F^2^. The threshold expression of F^2^ > 2sigma(F^2^) is used only for calculating R-factors(gt) etc. and is not relevant to the choice of reflections for refinement. R-factors based on F^2^ are statistically about twice as large as those based on F, and R- factors based on ALL data will be even larger.

### Fractional atomic coordinates and isotropic or equivalent isotropic displacement parameters (Å^2^)

**Table:**

	*x*	*y*	*z*	*U*_iso_*/*U*_eq_	
Zn1	0.5000	0.74469 (2)	0.2500	0.04143 (13)	
O1	0.41787 (9)	0.77620 (12)	0.4394 (2)	0.0656 (5)	
O2	0.44479 (7)	0.65455 (11)	0.33298 (16)	0.0544 (4)	
O3	0.57370 (8)	0.99041 (10)	0.66283 (17)	0.0589 (4)	
O4	0.5000	0.88159 (15)	0.2500	0.0502 (5)	
H41	0.5262 (12)	0.9122 (18)	0.222 (3)	0.057 (8)*	
N1	0.55509 (9)	0.75557 (11)	0.4431 (2)	0.0449 (4)	
N2	0.58656 (10)	0.91889 (14)	0.8743 (2)	0.0522 (5)	
H21	0.5893 (13)	0.867 (2)	0.924 (3)	0.078*	
H22	0.5842 (13)	0.969 (2)	0.920 (3)	0.078*	
C1	0.41195 (9)	0.69614 (15)	0.4166 (2)	0.0436 (5)	
C2	0.36403 (9)	0.64607 (14)	0.4853 (2)	0.0433 (5)	
C3	0.30732 (11)	0.6708 (2)	0.4566 (3)	0.0657 (7)	
C4	0.26398 (12)	0.6235 (3)	0.5224 (4)	0.0879 (10)	
H4	0.2263	0.6396	0.5041	0.106*	
C5	0.27421 (13)	0.5537 (3)	0.6139 (4)	0.0834 (10)	
C6	0.33025 (13)	0.53126 (19)	0.6414 (3)	0.0664 (7)	
H6	0.3379	0.4846	0.7038	0.080*	
C7	0.37565 (10)	0.57607 (14)	0.5789 (2)	0.0481 (5)	
C8	0.43568 (12)	0.5514 (2)	0.6170 (3)	0.0692 (7)	
H8A	0.4354	0.5117	0.6980	0.104*	
H8B	0.4568	0.6035	0.6420	0.104*	
H8C	0.4534	0.5231	0.5356	0.104*	
C9	0.29388 (16)	0.7462 (3)	0.3543 (5)	0.1093 (15)	
H9A	0.2533	0.7555	0.3514	0.164*	
H9B	0.3074	0.7319	0.2589	0.164*	
H9C	0.3124	0.7988	0.3877	0.164*	
C10	0.22443 (18)	0.5042 (4)	0.6832 (6)	0.144 (2)	
H10A	0.1901	0.5384	0.6732	0.217*	
H10B	0.2323	0.4949	0.7840	0.217*	
H10C	0.2196	0.4484	0.6358	0.217*	
C11	0.55380 (9)	0.82679 (13)	0.5282 (2)	0.0431 (5)	
H11	0.5294	0.8726	0.5026	0.052*	
C12	0.58642 (9)	0.83658 (13)	0.6518 (2)	0.0396 (4)	
C13	0.62260 (11)	0.76826 (16)	0.6900 (3)	0.0521 (5)	
H13	0.6449	0.7718	0.7732	0.063*	
C14	0.62482 (12)	0.69502 (16)	0.6023 (3)	0.0605 (7)	
H14	0.6492	0.6485	0.6248	0.073*	
C15	0.59098 (11)	0.69098 (15)	0.4816 (2)	0.0524 (6)	
H15	0.5930	0.6410	0.4235	0.063*	
C16	0.58181 (9)	0.92186 (14)	0.7316 (2)	0.0427 (5)	

### Atomic displacement parameters (Å^2^)

**Table:**

	*U*^11^	*U*^22^	*U*^33^	*U*^12^	*U*^13^	*U*^23^
Zn1	0.0472 (2)	0.02853 (19)	0.0485 (2)	0.000	0.00026 (14)	0.000
O1	0.0772 (12)	0.0463 (10)	0.0733 (12)	−0.0103 (9)	0.0122 (10)	−0.0028 (9)
O2	0.0569 (9)	0.0608 (10)	0.0456 (8)	−0.0090 (8)	0.0146 (7)	−0.0075 (7)
O3	0.0898 (12)	0.0369 (8)	0.0499 (9)	0.0059 (8)	0.0028 (8)	0.0006 (7)
O4	0.0559 (14)	0.0318 (11)	0.0630 (14)	0.000	0.0037 (11)	0.000
N1	0.0525 (11)	0.0331 (9)	0.0490 (10)	0.0057 (7)	−0.0026 (8)	−0.0027 (7)
N2	0.0718 (13)	0.0428 (11)	0.0422 (10)	−0.0075 (9)	0.0027 (9)	−0.0030 (8)
C1	0.0462 (11)	0.0490 (12)	0.0355 (9)	−0.0037 (9)	0.0004 (8)	0.0012 (8)
C2	0.0450 (11)	0.0459 (11)	0.0389 (10)	−0.0030 (9)	0.0039 (8)	−0.0067 (8)
C3	0.0488 (13)	0.087 (2)	0.0613 (15)	0.0026 (13)	−0.0020 (11)	0.0045 (14)
C4	0.0415 (14)	0.135 (3)	0.088 (2)	−0.0110 (17)	0.0016 (13)	0.002 (2)
C5	0.0654 (18)	0.105 (3)	0.080 (2)	−0.0333 (17)	0.0173 (15)	−0.0014 (19)
C6	0.0767 (18)	0.0598 (16)	0.0626 (15)	−0.0218 (13)	0.0108 (13)	0.0010 (12)
C7	0.0555 (13)	0.0386 (11)	0.0501 (11)	−0.0058 (9)	0.0068 (10)	−0.0057 (9)
C8	0.0644 (16)	0.0619 (16)	0.0814 (18)	0.0082 (13)	0.0039 (14)	0.0188 (14)
C9	0.070 (2)	0.142 (4)	0.116 (3)	0.021 (2)	−0.014 (2)	0.046 (3)
C10	0.091 (3)	0.186 (5)	0.157 (4)	−0.069 (3)	0.034 (3)	0.024 (4)
C11	0.0447 (11)	0.0342 (10)	0.0506 (11)	0.0086 (8)	−0.0034 (9)	−0.0035 (8)
C12	0.0422 (10)	0.0376 (10)	0.0391 (9)	0.0020 (8)	0.0046 (8)	0.0031 (8)
C13	0.0596 (14)	0.0548 (13)	0.0420 (11)	0.0116 (11)	−0.0022 (10)	0.0076 (10)
C14	0.0782 (17)	0.0474 (13)	0.0559 (13)	0.0274 (12)	−0.0024 (12)	0.0056 (10)
C15	0.0722 (15)	0.0354 (11)	0.0497 (12)	0.0134 (10)	0.0015 (11)	−0.0015 (9)
C16	0.0467 (11)	0.0384 (11)	0.0431 (10)	−0.0022 (8)	0.0034 (8)	−0.0006 (8)

### Geometric parameters (Å, º)

**Table:**

Zn1—O2^i^	2.0311 (16)	C6—C5	1.379 (5)
Zn1—O2	2.0311 (16)	C6—H6	0.9300
Zn1—O4	2.076 (2)	C7—C6	1.387 (3)
Zn1—N1	2.2066 (19)	C7—C8	1.496 (4)
Zn1—N1^i^	2.2066 (19)	C8—H8A	0.9600
O1—C1	1.240 (3)	C8—H8B	0.9600
O2—C1	1.259 (3)	C8—H8C	0.9600
O3—C16	1.233 (3)	C9—H9A	0.9600
O4—H41	0.81 (3)	C9—H9B	0.9600
N1—C11	1.336 (3)	C9—H9C	0.9600
N1—C15	1.338 (3)	C10—H10A	0.9600
N2—C16	1.324 (3)	C10—H10B	0.9600
N2—H21	0.91 (3)	C10—H10C	0.9600
N2—H22	0.87 (3)	C11—H11	0.9300
C2—C1	1.496 (3)	C12—C11	1.382 (3)
C2—C3	1.404 (3)	C12—C13	1.384 (3)
C2—C7	1.396 (3)	C12—C16	1.493 (3)
C3—C4	1.383 (4)	C13—C14	1.376 (3)
C3—C9	1.516 (4)	C13—H13	0.9300
C4—H4	0.9300	C14—H14	0.9300
C5—C4	1.375 (5)	C15—C14	1.369 (3)
C5—C10	1.526 (4)	C15—H15	0.9300
			
O2^i^—Zn1—O2	95.38 (9)	C6—C7—C8	119.9 (2)
O2—Zn1—O4	132.31 (5)	C7—C8—H8A	109.5
O2^i^—Zn1—O4	132.31 (5)	C7—C8—H8B	109.5
O2—Zn1—N1	96.72 (7)	C7—C8—H8C	109.5
O2^i^—Zn1—N1	89.07 (7)	H8A—C8—H8B	109.5
O2—Zn1—N1^i^	89.07 (7)	H8A—C8—H8C	109.5
O2^i^—Zn1—N1^i^	96.72 (7)	H8B—C8—H8C	109.5
O4—Zn1—N1	85.71 (4)	C3—C9—H9A	109.5
O4—Zn1—N1^i^	85.71 (4)	C3—C9—H9B	109.5
N1—Zn1—N1^i^	171.42 (8)	C3—C9—H9C	109.5
C1—O2—Zn1	106.41 (14)	H9A—C9—H9B	109.5
Zn1—O4—H41	125 (2)	H9A—C9—H9C	109.5
C11—N1—Zn1	121.50 (14)	H9B—C9—H9C	109.5
C11—N1—C15	116.7 (2)	C5—C10—H10A	109.5
C15—N1—Zn1	121.76 (15)	C5—C10—H10B	109.5
C16—N2—H21	122.6 (19)	C5—C10—H10C	109.5
C16—N2—H22	117 (2)	H10A—C10—H10B	109.5
H21—N2—H22	121 (3)	H10A—C10—H10C	109.5
O1—C1—O2	121.8 (2)	H10B—C10—H10C	109.5
O1—C1—C2	120.6 (2)	N1—C11—C12	124.08 (19)
O2—C1—C2	117.6 (2)	N1—C11—H11	118.0
C3—C2—C1	119.5 (2)	C12—C11—H11	118.0
C7—C2—C1	120.20 (19)	C11—C12—C13	117.9 (2)
C7—C2—C3	120.3 (2)	C11—C12—C16	117.47 (18)
C2—C3—C9	121.0 (3)	C13—C12—C16	124.6 (2)
C4—C3—C2	118.1 (3)	C12—C13—H13	120.7
C4—C3—C9	120.9 (3)	C14—C13—C12	118.5 (2)
C3—C4—H4	118.6	C14—C13—H13	120.7
C5—C4—C3	122.8 (3)	C13—C14—H14	120.2
C5—C4—H4	118.6	C15—C14—C13	119.6 (2)
C4—C5—C6	118.0 (3)	C15—C14—H14	120.2
C4—C5—C10	120.2 (4)	N1—C15—C14	123.1 (2)
C6—C5—C10	121.8 (4)	N1—C15—H15	118.4
C5—C6—C7	122.0 (3)	C14—C15—H15	118.4
C5—C6—H6	119.0	O3—C16—N2	123.7 (2)
C7—C6—H6	119.0	O3—C16—C12	119.17 (18)
C2—C7—C8	121.3 (2)	N2—C16—C12	117.14 (19)
C6—C7—C2	118.8 (2)		
			
O2^i^—Zn1—O2—C1	166.98 (17)	C7—C2—C3—C9	−179.5 (3)
O4—Zn1—O2—C1	−13.02 (17)	C1—C2—C7—C6	179.7 (2)
N1—Zn1—O2—C1	77.29 (14)	C1—C2—C7—C8	2.2 (3)
N1^i^—Zn1—O2—C1	−96.36 (14)	C3—C2—C7—C6	0.6 (3)
O2—Zn1—N1—C11	−106.95 (18)	C3—C2—C7—C8	−176.9 (2)
O2^i^—Zn1—N1—C11	157.75 (18)	C2—C3—C4—C5	−0.2 (5)
O2—Zn1—N1—C15	72.40 (19)	C9—C3—C4—C5	178.8 (4)
O2^i^—Zn1—N1—C15	−22.91 (19)	C6—C5—C4—C3	0.7 (6)
O4—Zn1—N1—C11	25.19 (17)	C10—C5—C4—C3	180.0 (4)
O4—Zn1—N1—C15	−155.46 (19)	C7—C6—C5—C4	−0.7 (5)
Zn1—O2—C1—O1	−2.4 (3)	C7—C6—C5—C10	−179.9 (3)
Zn1—O2—C1—C2	175.91 (15)	C2—C7—C6—C5	0.1 (4)
Zn1—N1—C11—C12	178.69 (16)	C8—C7—C6—C5	177.5 (3)
C15—N1—C11—C12	−0.7 (3)	C13—C12—C11—N1	−0.1 (3)
Zn1—N1—C15—C14	−178.6 (2)	C16—C12—C11—N1	177.5 (2)
C11—N1—C15—C14	0.8 (4)	C11—C12—C13—C14	0.9 (3)
C3—C2—C1—O1	60.0 (3)	C16—C12—C13—C14	−176.5 (2)
C3—C2—C1—O2	−118.4 (2)	C11—C12—C16—O3	−33.6 (3)
C7—C2—C1—O1	−119.1 (2)	C11—C12—C16—N2	146.1 (2)
C7—C2—C1—O2	62.5 (3)	C13—C12—C16—O3	143.8 (2)
C1—C2—C3—C4	−179.6 (3)	C13—C12—C16—N2	−36.4 (3)
C1—C2—C3—C9	1.4 (4)	C12—C13—C14—C15	−0.9 (4)
C7—C2—C3—C4	−0.5 (4)	N1—C15—C14—C13	0.0 (4)

Symmetry code: (i) −*x*+1, *y*, −*z*+1/2.

### Hydrogen-bond geometry (Å, º)

**Table:**

*D*—H···*A*	*D*—H	H···*A*	*D*···*A*	*D*—H···*A*
N2—H21···O1^ii^	0.91 (3)	1.88 (3)	2.766 (3)	165 (3)
N2—H22···O3^iii^	0.87 (3)	2.34 (3)	3.013 (2)	134 (2)
O4—H41···O3^iv^	0.81 (3)	1.93 (3)	2.719 (2)	165 (3)

Symmetry codes: (ii) −*x*+1, *y*, −*z*+3/2; (iii) *x*, −*y*+2, *z*+1/2; (iv) *x*, −*y*+2, *z*−1/2.
