# Supplementary figures and images for: Crystal structure and Hirshfeld surface analysis of aqua­bis­(nicotinamide-κN 1)bis­(2,4,6-tri­methyl­benzoato-κO)zinc
Source: Acta Crystallogr E Crystallogr Commun. 2017 Aug 21;73(Pt 9):1348–52. doi: 10.1107/S2056989017011690 (PMC5588578; doi:10.1107/S2056989017011690)

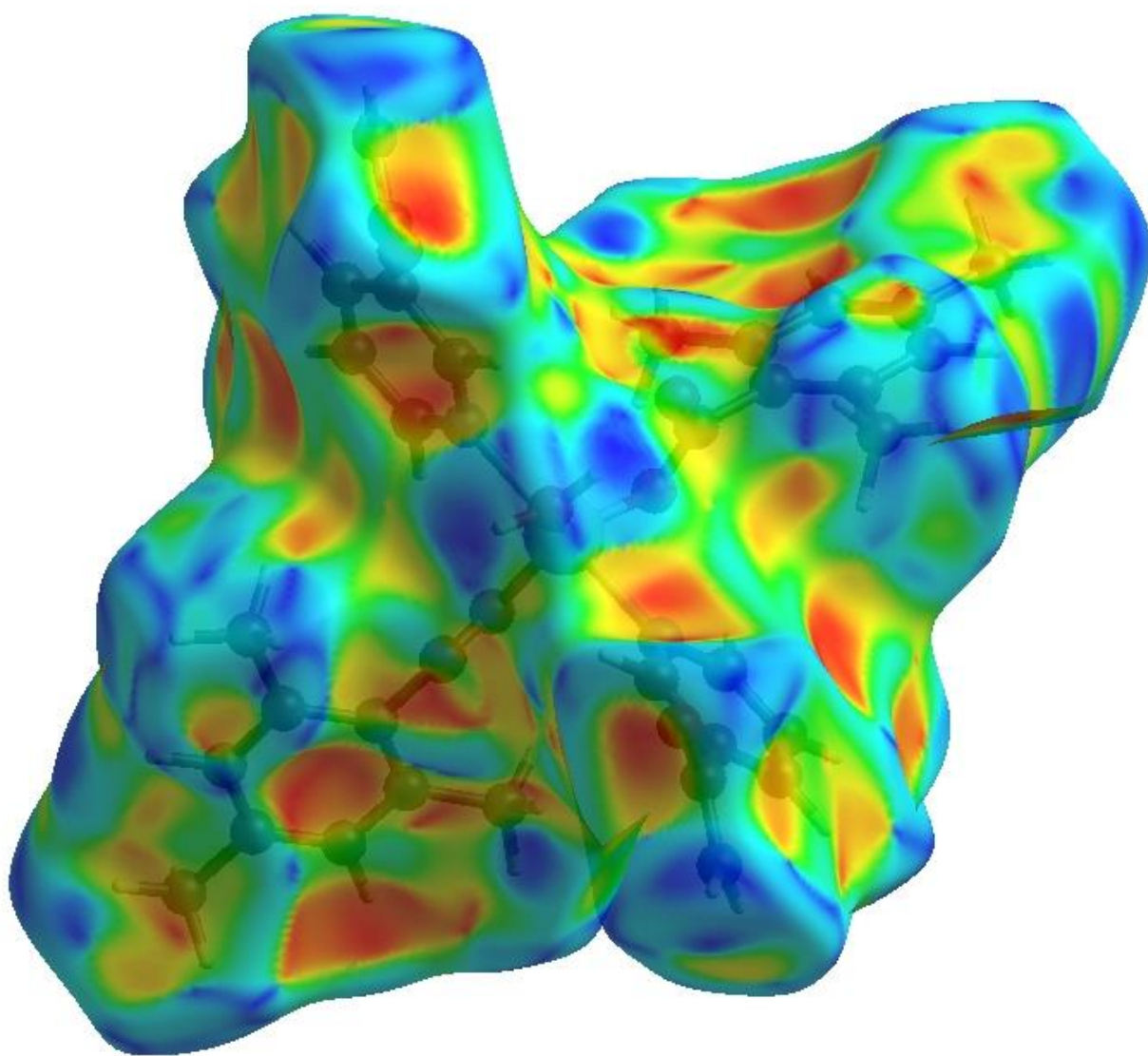

Figure s1

Hirshfeld surface of the title complex plotted over shape-index.

Supplement: Supplementary file 3 [file e-73-01348-sup3.pdf]

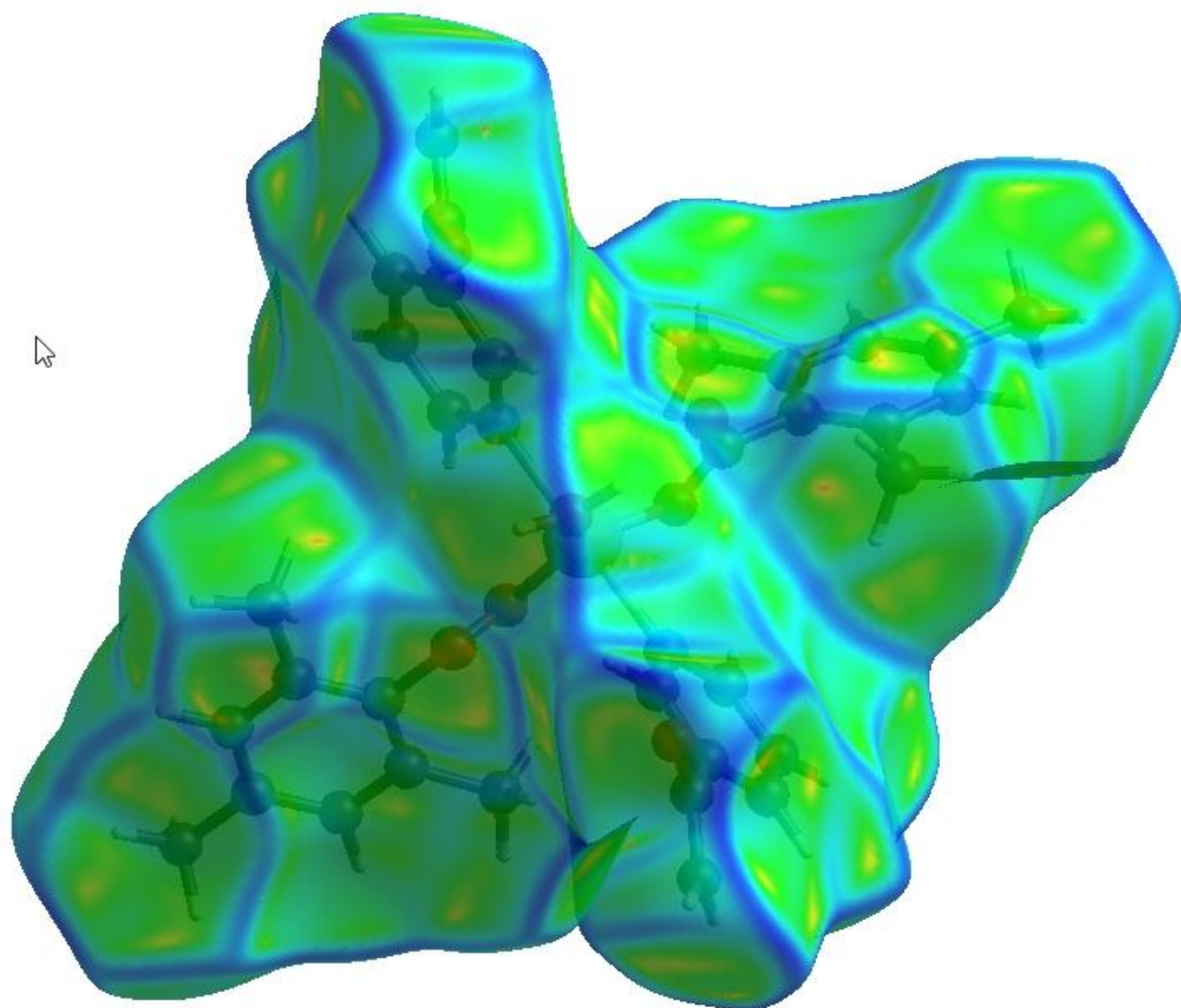

Figure s2

Hirshfeld surface of the title complex plotted over curvedness.

Supplement: Supplementary file 4 [file e-73-01348-sup4.pdf]
