# Supplementary material for: Crystal structure and Hirshfeld surface analysis of aqua­bis­(nicotinamide-κN 1)bis­(2,4,6-tri­methyl­benzoato-κO)zinc
Source: Acta Crystallogr E Crystallogr Commun. 2017 Aug 21;73(Pt 9):1348–52. doi: 10.1107/S2056989017011690 (PMC5588578; doi:10.1107/S2056989017011690)
Supplement: Supplementary file 5 [file e-73-01348-sup5.pdf]

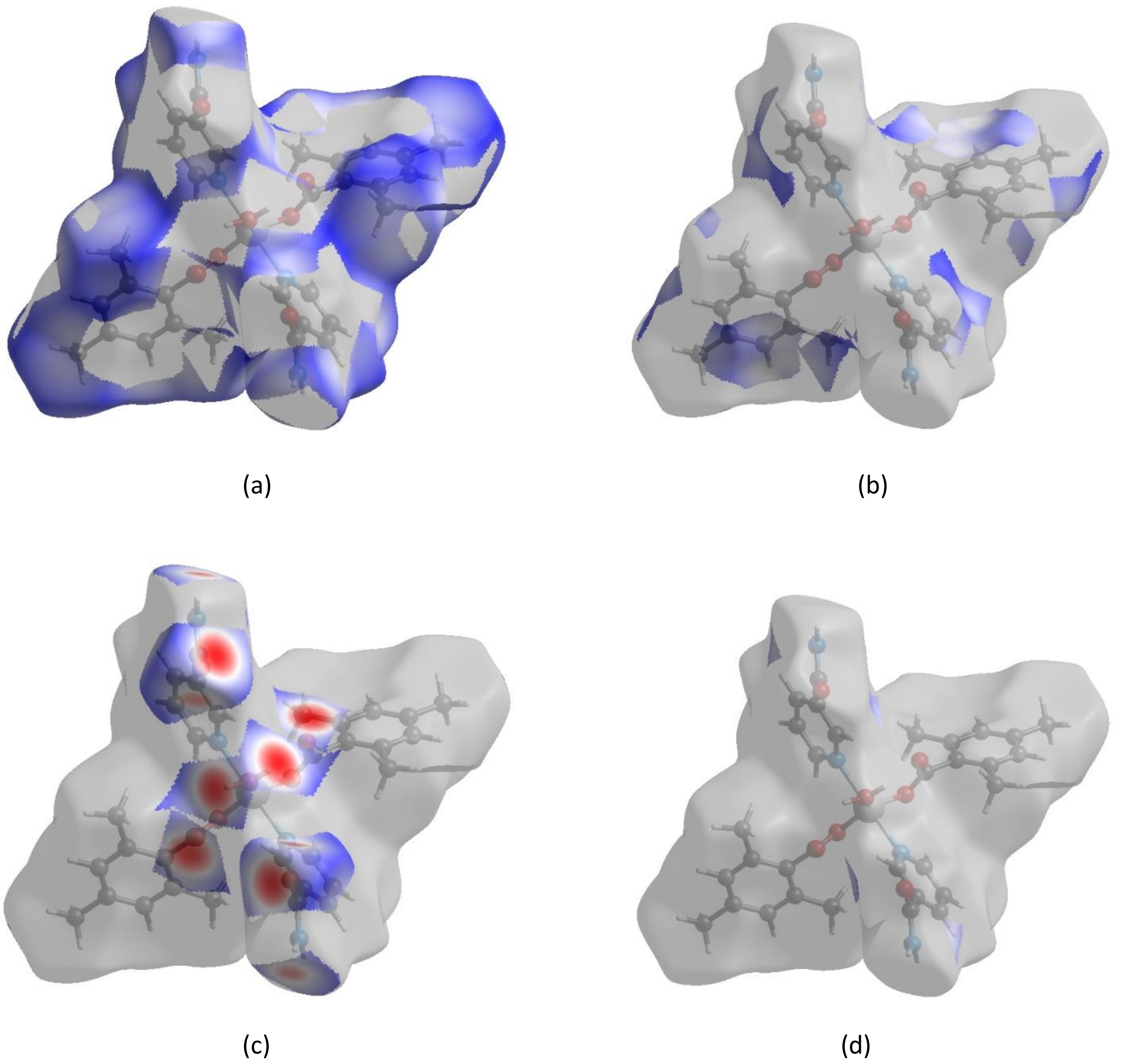

Figure s3

Hirshfeld surface representations with the function d_{norm} plotted onto the surface for (a) H...H, (b) H...C/C...H, (c) H...O/O...H and (d) H...N/N...H interactions.
